# Cognitive Impairment in Asthma: Emerging Evidence, Clinical Consequences, and Future Directions

**DOI:** 10.3390/jcm15197761

**Published:** 2026-10-08

**Authors:** Aminah Mengash, Rayan A. Siraj

**Affiliations:** Department of Respiratory Therapy, College of Applied Medical Sciences, King Faisal University, Al-Ahsa 31982, Saudi Arabia; rsiraj@kfu.edu.sa

**Keywords:** asthma, cognitive impairment, cognitive decline, neuroinflammation

## Abstract

Asthma is increasingly recognised as a systemic inflammatory disease with effects extending beyond the respiratory system. Emerging evidence suggests that cognitive impairment may represent an under-recognised extrapulmonary manifestation with important implications for disease management and long-term neurological health. This narrative review critically synthesises current evidence regarding the epidemiology, clinical consequences, and potential mechanisms linking asthma with cognitive dysfunction. Observational and longitudinal studies indicate that asthma is associated with an increased risk of cognitive impairment, cognitive decline, and dementia. However, substantial heterogeneity in study design, cognitive assessment, asthma phenotyping, and adjustment for confounding factors limits causal inference. Cognitive deficits predominantly involve attention, executive function, memory, and, less consistently, processing speed, potentially compromising medication adherence, inhaler technique, symptom perception, and self-management. Current evidence supports a multifactorial pathophysiological model in which chronic systemic inflammation, oxidative stress, intermittent hypoxaemia, sleep disturbance, corticosteroid exposure, and psychological comorbidity interact to promote endothelial dysfunction, blood–brain barrier disruption, microglial activation, neuroinflammation, mitochondrial dysfunction, and impaired synaptic plasticity. These mechanisms may be particularly relevant in children with early-onset asthma, older adults, and patients with severe or poorly controlled disease, although direct mechanistic evidence remains limited. Future research should prioritise well-phenotyped longitudinal cohorts that integrate comprehensive cognitive assessment with neuroimaging, inflammatory biomarkers, and objective measures of disease control to establish temporal relationships, identify high-risk populations, and determine whether cognitive dysfunction is a modifiable component of asthma burden. Recognition of cognition as a clinically relevant outcome may ultimately improve risk stratification and support more personalised, holistic approaches to asthma care.

## 1. Introduction

Asthma is a common chronic inflammatory disease of the airways characterised by variable respiratory symptoms, airway hyperresponsiveness, and reversible airflow limitation. It affects more than 200 million people worldwide, and its prevalence continues to increase in many regions, contributing substantially to global morbidity, healthcare utilisation, and impaired quality of life [1,2]. According to the Global Asthma Report 2022, asthma ranks among the leading causes of disease burden worldwide [1]. In Saudi Arabia, the prevalence of physician-diagnosed asthma among adults has increased from approximately 4% to 11% over recent decades, reflecting the growing public health impact of the disease [1,2]. Beyond its clinical consequences, asthma is associated with considerable socioeconomic burden through increased healthcare costs, reduced productivity, and recurrent healthcare utilisation.

Although asthma has traditionally been regarded as a respiratory disorder, increasing evidence suggests that it is a complex, multi-dimensional disease with effects that extend beyond the lungs [3]. A defining feature of asthma is its frequent coexistence with a range of comorbid conditions, including allergic rhinitis, gastro-oesophageal reflux disease, obesity, obstructive sleep apnoea, and psychological disorders such as anxiety and depression [3,4]. These comorbidities may share common pathophysiological pathways with asthma or develop independently; however, their presence can complicate disease management, contribute to poorer asthma control, and increase healthcare utilisation. Collectively, they reflect a more complex and vulnerable clinical phenotype.

In recent years, growing attention has been directed toward the potential effects of asthma on brain health. Experimental and clinical evidence has suggested the existence of a lung–brain axis, whereby chronic airway inflammation, immune activation, and intermittent reductions in oxygenation may influence neurological function and cognitive processes [5,6]. Neuroimaging studies have reported structural and functional brain changes among individuals with asthma, providing further support for a potential relationship between respiratory disease and brain health [7,8,9]. Although the precise mechanisms remain incompletely understood, these observations have generated increasing interest in the broader neurological and psychological consequences of asthma.

The relationship between asthma and mental health has been more extensively studied than its relationship with cognition. Numerous studies have reported higher rates of anxiety and depression among individuals with asthma, particularly those with persistent or poorly controlled disease [10,11]. Importantly, these psychological comorbidities have been associated with poorer asthma control, reduced quality of life, and increased disease burden. Given that anxiety, depression, and sleep disturbances are themselves associated with cognitive dysfunction, it is plausible that asthma-related cognitive impairment may arise through a combination of direct disease-related mechanisms and indirect effects mediated by common comorbid conditions.

Within this context, cognitive impairment represents a potentially important but relatively underexplored dimension of asthma burden. Cognitive function plays a central role in effective asthma self-management, requiring patients to recognise symptoms, adhere to treatment regimens, use inhaler devices correctly, and respond appropriately to changes in their condition. Emerging evidence suggests that asthma may be associated with reduced cognitive performance across several domains and may increase the risk of mild cognitive impairment, dementia, and Alzheimer’s disease [12,13,14]. Furthermore, longer asthma duration, impaired lung function, and greater disease burden have been associated with poorer cognitive outcomes [15,16]. Neuroimaging studies have also reported smaller hippocampal volumes among individuals with asthma, which may contribute to cognitive dysfunction [17].

Despite these observations, the relationship between asthma and cognitive impairment remains poorly understood. Existing studies vary considerably in design, populations, cognitive assessment methods, and outcome definitions, making comparisons difficult and limiting the ability to draw definitive conclusions. Cognitive outcomes are often evaluated as secondary measures, and the extent to which cognitive impairment reflects asthma-related pathophysiology, disease severity, ageing, or the influence of comorbid conditions remains uncertain. Consequently, cognitive impairment remains largely overlooked in routine asthma care despite its potential implications for self-management, treatment adherence, and long-term health outcomes [18].

Accordingly, this narrative review aims to provide a comprehensive synthesis of the current evidence regarding cognitive impairment in asthma. Specifically, it examines the prevalence of cognitive dysfunction, explores its potential clinical consequences and underlying mechanisms, and critically appraises the limitations of the existing literature. By identifying key evidence gaps and areas for future research, this review seeks to clarify whether cognitive impairment represents an underrecognized component of asthma burden and an important consideration in the holistic management of individuals with asthma.

## 2. Methods and Materials

### Literature Search and Review Strategy

This narrative review was designed to synthesis the available evidence on the epidemiology, clinical consequences, and potential mechanisms linking asthma with cognitive impairment. Relevant literature was identified primarily through PubMed, Scopus, and Google Scholar using combination terms, including “asthma,” “cognitive impairment,” “cognitive decline,” “dementia,” “neuroinflammation,” “hypoxia,” and “corticosteroids.” Relevant original studies and review articles addressing the prevalence and longitudinal evidence, clinical consequences, and potential mechanisms of cognitive impairment in asthma were considered. Attention was given to studies examining cognitive outcomes across different age groups and asthma population.

## 3. Definition and Consideration

### 3.1. Cognition and Cognitive Impairment

Cognition encompasses a range of mental processes that enable individuals to acquire, process, retain, and utilise information to adapt effectively to their environment [19]. These processes are fundamental to daily functioning and include learning, thinking, reasoning, memory, language, attention, executive functioning, motor function, and emotional processing [13]. Together, these cognitive domains influence an individual’s ability to perform everyday activities, make decisions, solve problems, and maintain functional independence [20].

Understanding cognitive function has become increasingly important given the growing global burden of cognitive decline and dementia. Cognitive disorders represent a major public health challenge worldwide, affecting millions of individuals and placing considerable demands on families, healthcare systems, and society [21]. It has been estimated that approximately 50 million people were living with dementia globally in 2020, with projections suggesting that this number may nearly triple by 2050 [22,23]. In addition to their substantial impact on quality of life and functional independence, cognitive disorders are associated with considerable economic consequences, with the global cost of dementia exceeding one trillion US dollars annually [22]. These trends have intensified interest in identifying risk factors and clinical conditions that may contribute to cognitive decline and potentially increase the risk of future cognitive impairment.

Cognitive impairment is characterised by a decline or dysfunction in one or more cognitive domains, including memory, attention, and executive functioning [24]. The degree of impairment may vary from subtle deficits to more severe dysfunction that interferes with daily activities and independence. Mild cognitive impairment (MCI) is generally considered an intermediate stage between normal cognitive ageing and dementia and is characterised by measurable cognitive decline that is less likely to affect functional independence [25] substantially. Current evidence suggests that while MCI may represent a transitional state between normal ageing and dementia, not all individuals with MCI progress to dementia [24,25]. Among individuals with MCI, memory is often the most commonly affected cognitive domain, although impairments in other domains may also occur. Some individuals may experience subjective memory decline, referring to personal concerns regarding memory performance despite relatively preserved daily functioning [24]. Importantly, MCI may be reversible in some cases, and a proportion of affected individuals have been reported to return to normal cognitive functioning over time. Therefore, identifying factors associated with cognitive impairment, particularly modifiable risk factors, remains an important area of research for preventing progression to irreversible cognitive decline and dementia.

The underlying causes of cognitive impairment remain incompletely understood. However, several factors have been implicated in its development, including advancing age, smoking, diabetes mellitus, vascular disease, physical inactivity, infection, educational attainment, and genetic susceptibility [12].

### 3.2. Assessment of Cognitive Function

Assessment of cognitive function is an important consideration when interpreting the literature on cognitive impairment. A variety of validated instruments have been developed to screen for and assess cognitive dysfunction, ranging from brief screening tools to more comprehensive neuropsychological assessments [26]. These tools differ in the cognitive domains evaluated, the administration procedures, the scoring systems, and their sensitivity for detecting impairment. Consequently, the choice of assessment tool may influence the identification and classification of cognitive impairment within both clinical and research settings. A summary of commonly used cognitive assessment tools is presented in Table 1.

Among the most widely used cognitive screening instruments are the Mini-Mental State Examination (MMSE) and the Montreal Cognitive Assessment (MoCA) [26,27]. While the MMSE has historically been one of the most commonly used tools for cognitive screening, the MoCA has demonstrated greater sensitivity for detecting mild cognitive impairment and is frequently used in both clinical and research settings [27]. Notably, studies investigating cognitive impairment in asthma have commonly utilised the MoCA to assess cognitive function. This may be particularly relevant given that mild cognitive impairment, rather than overt dementia, is the outcome most frequently examined in the asthma literature.

Several other validated instruments are available, including the Addenbrooke’s Cognitive Examination-Revised (ACE-R), Clock Drawing Test, Mini-Cog, Informant Questionnaire on Cognitive Decline in the Elderly (IQCODE), Cambridge Cognitive Examination (CAMCOG), Quick Cognitive Screening Test (QCST), and Memory and Executive Screening (MES) [27,28,29,30,31,32,33,34]. More recently, technology-based approaches, including virtual reality cognitive assessments, have also been explored [35]. Although these tools assess similar constructs, they vary in the cognitive domains emphasised and the thresholds used to define impairment (Table 1).

This variation in assessment methods represents an important source of methodological heterogeneity within the existing asthma literature. Some studies have relied on brief screening tools, whereas others have used more comprehensive cognitive assessments. Furthermore, cognitive impairment may be defined differently across studies, making direct comparisons challenging. As a result, differences in the reported prevalence and severity of cognitive impairment may partly reflect methodological differences rather than true variations in cognitive function among individuals with asthma.

Another important consideration is that most available cognitive screening tools were developed for general populations rather than specifically for individuals with asthma. Consequently, they may not fully capture the particular cognitive challenges that could be associated with asthma-related factors such as symptom burden, sleep disturbance, psychological comorbidities, or disease control. While currently available tools provide a practical approach for assessing cognitive function, the absence of standardised assessment methods across asthma studies limits comparability and may contribute to inconsistencies in the literature. Therefore, when interpreting findings related to cognitive impairment in asthma, consideration should be given to the assessment instrument used and its potential influence on study outcomes.

## 4. Prevalence of Cognitive Impairment in Asthma

The prevalence of cognitive impairment among individuals with asthma remains uncertain and appears to vary considerably across studies. Differences in study populations, age distributions, disease severity, comorbidities, and cognitive assessment methods have contributed to substantial variability in reported estimates. Nevertheless, emerging evidence suggests that cognitive dysfunction may represent an important but under-recognised component of asthma burden.

The most comprehensive evidence to date comes from a recent systematic review and meta-analysis by Bushi et al. (2025) [18], which included 12 studies, of which 10 were eligible for quantitative synthesis. The authors reported a pooled prevalence of cognitive impairment of 16.3% among individuals with asthma and found that asthma was associated with a significantly increased risk of cognitive impairment compared with individuals without asthma (HR = 1.47, 95% CI: 1.09–1.84). Furthermore, the review identified an increased risk of cognitive decline, dementia, and Alzheimer’s disease among patients with asthma, suggesting that the cognitive consequences of asthma may extend beyond mild cognitive dysfunction. However, substantial heterogeneity and evidence of publication bias were noted across the included studies, limiting the certainty of the pooled estimates. 

Individual studies have reported considerably higher prevalence estimates than those observed in pooled analyses. In a retrospective study involving 202 adults with asthma, Rhyou and Nam (2021) [16] reported that 44.1% of participants exhibited cognitive impairment based on the Montreal Cognitive Assessment (MoCA). Cognitive impairment was substantially more common among older adults, affecting 72.9% of patients aged 65 years and older compared with 23.1% of those younger than 65 years. In the same study, lower lung function was associated with poorer cognitive performance, while longer asthma duration was independently associated with cognitive impairment, suggesting that cumulative disease burden may contribute to cognitive decline over time. Interestingly, asthma severity itself was not significantly associated with cognitive performance, indicating that factors beyond traditional measures of disease severity may influence cognitive outcomes.

Similar observations have been reported in Middle Eastern populations. In a cross-sectional study conducted among middle-aged and elderly individuals in Saudi Arabia, Abuaish et al. (2023) [36] found that asthma was associated with an increased likelihood of mild cognitive impairment after adjustment for potential confounders (OR = 1.80, 95% CI: 1.00–3.24). Notably, age-stratified analyses demonstrated that elderly individuals with asthma had substantially greater odds of mild cognitive impairment than their non-asthmatic counterparts (OR = 7.4, 95% CI: 2.34–23.31). However, the same study also reported significant associations between asthma and symptoms of anxiety and depression, suggesting that psychological comorbidities may contribute to the observed cognitive burden. These findings highlight the difficulty of distinguishing the independent effects of asthma from those of coexisting conditions that are themselves associated with impaired cognitive function.

Interpretation of the prevalence literature is further complicated by methodological differences across studies. Cognitive impairment has been assessed using a variety of screening and neuropsychological instruments, including the MoCA and other cognitive assessment tools, which differ in their sensitivity, specificity, and ability to detect deficits across cognitive domains [16,18]. Moreover, many studies have focused predominantly on older adults, who are inherently at greater risk of cognitive decline, while adjustment for important confounders such as depression, anxiety, sleep disorders, cardiovascular disease, and educational attainment has been inconsistent [18,36]. Consequently, it remains unclear to what extent the reported cognitive deficits are directly attributable to asthma itself or reflect the broader burden of ageing and comorbidity.

Taken together, the available evidence suggests that cognitive impairment may affect a substantial proportion of individuals with asthma, particularly older adults and those with longer disease duration or impaired lung function [16,36]. However, considerable heterogeneity exists across studies, and prevalence estimates appear to be highly dependent on study design, participant characteristics, and the cognitive assessment tools employed [18]. Therefore, the true prevalence of cognitive impairment in asthma remains difficult to establish, highlighting the need for larger, methodologically robust studies using standardised cognitive assessments and adequate control of potential confounding factors.

## 5. Incidence and Longitudinal Evidence

### 5.1. Limited Longitudinal Evidence Assessing Incident Cognitive Decline

Although accumulating evidence suggests a link between asthma and impaired cognitive function, longitudinal studies examining incident cognitive decline remain limited. Consequently, it is unclear whether asthma contributes directly to progressive cognitive deterioration or simply coexists with established risk factors for cognitive impairment. A recent systematic review and meta-analysis reported that asthma was associated with a 47% higher risk of cognitive impairment [18]. Additional evidence suggested an increased risk of incident dementia among individuals with asthma [37]. However, the relatively small number of prospective studies, together with considerable between-study heterogeneity and possible publication bias, limits causal inference and underscores the need for large, well-characterised longitudinal cohorts to establish the temporal relationship between asthma and cognitive decline.

Emerging evidence further suggests that the cognitive consequences of asthma may begin early in life rather than being confined to older age. Meta-analytic data indicate that children with asthma exhibit significantly greater attention deficits than healthy peers, whereas memory function appears to be relatively preserved [38,39]. These findings raise the possibility that asthma-related cognitive alterations originate during neurodevelopment and may influence cognitive trajectories across the lifespan. However, because prospective paediatric studies remain limited, it is unknown whether these early deficits persist, progress, or resolve with improved disease control and maturation.

The clinical characteristics that predispose individuals with asthma to subsequent cognitive decline also remain poorly understood. Although preliminary evidence suggests that frequent exacerbations and asthma-related hospitalisations are associated with poorer cognitive performance [40], the relative contributions of disease severity, inflammatory phenotype, treatment exposure, and cumulative disease burden remain uncertain.

### 5.2. Emerging Longitudinal Evidence

Despite the limited number of longitudinal studies, emerging evidence increasingly suggests that asthma is associated with less favourable cognitive trajectories across the lifespan, as shown in Table 2. Using data from nine waves of the English Longitudinal Study of Ageing, one prospective analysis demonstrated that incident asthma was associated with accelerated memory decline following diagnosis, whereas cognitive trajectories before asthma onset were comparable with those of matched controls. These findings suggest that cognitive deterioration may develop after the onset of asthma rather than representing a pre-existing characteristic [41]. Similarly, analysis of the Adolescent Brain Cognitive Development cohort showed that children with earlier-onset asthma experienced slower improvement in episodic memory over two years, supporting the hypothesis that prolonged exposure to asthma during critical periods of brain development may alter normal cognitive maturation [42].

Evidence from older adults further supports an association between asthma and clinically meaningful neurocognitive outcomes. In a large US Medicare cohort, asthma was associated with a 20–33% higher two-year incidence of Alzheimer’s disease after demographic adjustment and matching [43]. Extending these epidemiological observations, one study reported that severe asthma was associated with cerebrospinal fluid biomarkers of synaptic degeneration together with greater longitudinal cognitive decline, particularly among individuals with additional cardiovascular or genetic susceptibility to dementia [21]. Together, these findings suggest that asthma severity and cumulative disease burden may amplify underlying neurodegenerative processes in susceptible individuals.

Asthma control may also influence cognitive outcomes, although the direction of this relationship remains uncertain. Among older adults with asthma, cognitive impairment defined using a conservative Montreal Cognitive Assessment threshold was associated with worse asthma control and poorer inhaled-corticosteroid adherence [44]. These observations support a potentially bidirectional relationship in which poorly controlled asthma contributes to cognitive vulnerability through persistent inflammation, recurrent exacerbations, sleep disturbance and intermittent hypoxaemia, while cognitive impairment simultaneously compromises treatment adherence, symptom recognition and effective self-management.

### 5.3. Challenges

**Figure 1:**
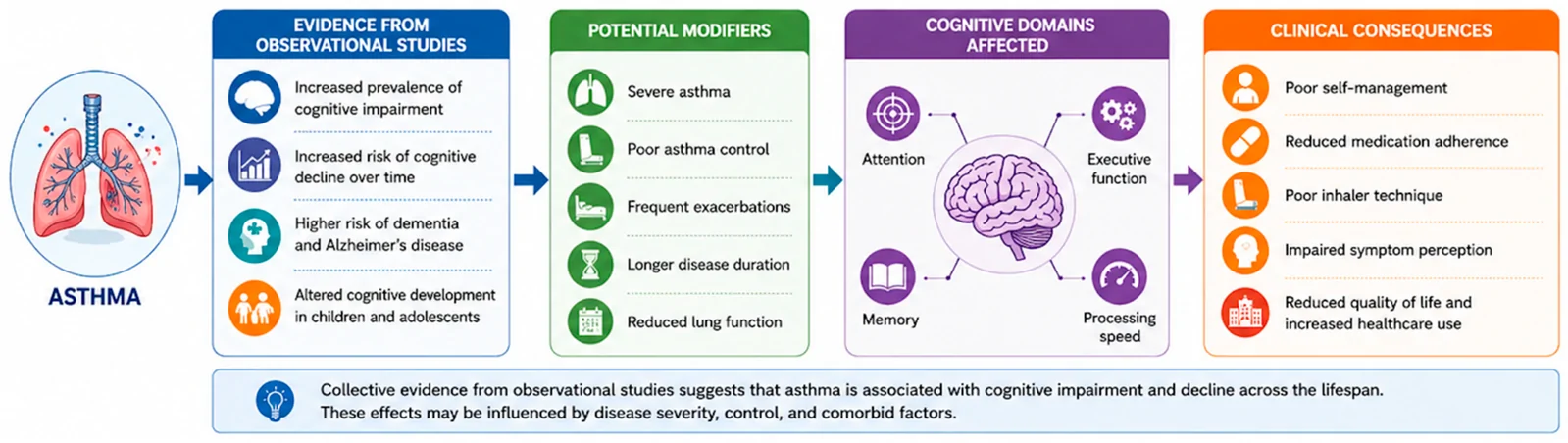
Evidence linking asthma and cognitive impairment. The figure was created with the assistance of ChatGPT (OpenAI; https://chatgpt.com) for visual presentation. [12,15,16,18,38,40,41,43,45,46,47,48].

Despite the growing body of evidence linking asthma with cognitive impairment, important methodological limitations continue to complicate interpretation of the available literature. Although observational studies consistently suggest an association between asthma and adverse cognitive outcomes across the lifespan, the current evidence remains heterogeneous and insufficient to establish a causal relationship. As illustrated in Figure 1, asthma has been associated with an increased prevalence of cognitive impairment, accelerated cognitive decline, and a higher risk of dementia, while disease severity, asthma control, exacerbation burden, and lung function influence these relationships. However, the strength and consistency of these associations differ substantially across studies.

Several factors contribute to this variability. First, substantial heterogeneity exists in study design, participant characteristics, and cognitive assessment methods. Studies have employed diverse screening instruments, neuropsychological tests, and outcome definitions, limited direct comparisons and contributing to variability in reported estimates. Second, most available evidence is derived from cross-sectional studies, whereas relatively few longitudinal investigations have evaluated incident cognitive decline, thereby limited conclusions regarding temporal sequence and causality. Third, potential confounding remains a major concern. Important factors including age, educational attainment, socioeconomic status, smoking, cardiovascular disease, obesity, depression, anxiety, and sleep disorders—have not been consistently accounted for, raising the possibility of residual confounding. Finally, asthma itself is a heterogeneous disease, yet few studies have adequately characterised disease severity, inflammatory phenotype, asthma control, exacerbation frequency, or treatment exposure. Consequently, it remains unclear which patient subgroups are at greatest risk of cognitive decline and whether the observed associations reflect asthma itself or the cumulative effects of disease burden and associated comorbidities.

#### 5.3.1. Heterogeneity in Study Design and Cognitive Assessment

The interpretation of cognitive outcomes in asthma is substantially complicated by marked heterogeneity in study design, participant characteristics, cognitive assessment methods, and definitions of cognitive impairment. Existing studies have been conducted in diverse populations, including children, community-dwelling older adults, and hospital-based cohorts, resulting in considerable variation in age, disease severity, comorbidity burden, and clinical setting [18,37,38]. For example, Ray et al. evaluated cognitive function in elderly adults with asthma, whereas Penchev et al. examined attention and memory in paediatric populations, illustrating the challenges of directly comparing cognitive outcomes across different age groups and stages of disease [15,39].

Methodological variability is further amplified by the use of different cognitive assessment instruments. Studies have employed a wide range of tools, including the Montreal Cognitive Assessment (MoCA), Mini-Mental State Examination (MMSE), dementia diagnostic codes, and comprehensive neuropsychological batteries, each differing in sensitivity, specificity, and the cognitive domains evaluated [49,50,51]. The MoCA, for example, has demonstrated greater sensitivity than the MMSE for detecting mild cognitive impairment, particularly among individuals with subtle cognitive deficits [52]. Moreover, a systematic review assessing the validity of cognitive screening instruments identified substantial clinical and methodological heterogeneity and was unable to recommend a single optimal screening tool for dementia or mild cognitive impairment, emphasising the lack of standardisation across studies [53].

The absence of a consistent definition of cognitive impairment represents an additional challenge. Across studies, cognitive impairment has been defined using screening test thresholds, clinical diagnoses of mild cognitive impairment (MCI), dementia diagnostic codes, or performance on comprehensive neuropsychological testing batteries [50,54]. This inconsistency is reflected in the recent systematic review and meta-analysis by Bushi et al., which identified variation in study design and outcome definitions as major contributors to between-study heterogeneity [18]. Collectively, these methodological differences limit direct comparison between studies, contribute to inconsistent estimates, and impede a clear understanding of the prevalence, determinants, and clinical significance of cognitive dysfunction in asthma.

#### 5.3.2. Limited Longitudinal Evidence and Inability to Infer Causality

A second major limitation of the current evidence base is the scarcity of longitudinal studies capable of establishing temporal relationships between asthma and cognitive decline. Most published studies are cross-sectional, providing only a snapshot of cognitive function and precluding conclusions regarding causality. Although these studies consistently report associations between asthma and poorer cognitive performance, they cannot determine whether asthma directly contributes to cognitive deterioration, whether impaired cognition precedes asthma diagnosis, or whether both conditions arise from shared biological or environmental risk factors [18,55,56].

Prospective cohort studies remain relatively few, and those that have examined incident cognitive decline or dementia vary considerably in study design, duration of follow-up, participant selection, and cognitive outcome definitions [37,40]. This variability limits the ability to compare findings across cohorts and to identify consistent temporal patterns. Consequently, the direction and magnitude of the relationship between asthma and long-term cognitive decline remain incompletely understood.

#### 5.3.3. Residual Confounding and Inconsistent Adjustment for Covariates

Residual confounding represents another important challenge when interpreting the association between asthma and cognitive impairment. Cognitive function is influenced by numerous demographic, socioeconomic, behavioural, and clinical factors, many of which are also closely linked to asthma and its outcomes. Failure to account consistently for these variables may therefore partly explain the associations observed between asthma and cognitive dysfunction.

Age is the strongest recognised risk factor for cognitive decline and dementia, and several studies have reported a higher prevalence of cognitive impairment among older adults with asthma [16]. Likewise, lower educational attainment and socioeconomic status are well-established determinants of poorer cognitive outcomes and may also influence access to healthcare, treatment adherence, and disease management. Health literacy represents another potentially important but understudied factor. Patients with asthma and COPD identify the ability to access, understand, and apply health information as fundamental to effective self-management [57]. However, whether impaired cognitive function contributes to reduced health literacy, or vice versa, has received little investigation in asthma populations.

Lifestyle factors may provide an additional source of residual confounding. Smoking and second-hand smoke exposure have been associated with both impaired respiratory health and accelerated cognitive decline. Evidence from the China Health and Retirement Longitudinal Study demonstrated that current smokers were significantly more likely to follow a rapid cognitive decline trajectory than non-smokers, even after adjustment for demographic, social, and health-related factors, highlighting smoking as an important and potentially modifiable determinant of cognitive ageing [58].

The high prevalence of comorbid conditions among individuals with asthma further complicates interpretation of the available evidence. Cardiovascular disease, obesity, depression, anxiety, and sleep disorders are common in asthma and are independently recognised risk factors for cognitive impairment and dementia in the general population [59]. However, relatively few studies have explored the extent to which these comorbidities mediate or modify the relationship between asthma and cognitive outcomes. For example, a recent multicentre study from Ethiopia identified depression as an independent factor associated with cognitive impairment among adults with asthma [55]. Similarly, a cross-sectional study conducted in Saudi Arabia demonstrated associations between asthma, mild cognitive impairment, and symptoms of anxiety and depression, with elderly individuals with asthma exhibiting substantially higher odds of mild cognitive impairment [36]. Taken together, these findings suggest that cognitive impairment in asthma is likely to arise from multiple interacting mechanisms rather than asthma alone, although the relative contribution of individual comorbidities remains to be established.

#### 5.3.4. Limited Asthma Phenotyping and Identification of High-Risk Groups

The heterogeneous nature of asthma presents an additional challenge when interpreting cognitive outcomes. Asthma comprises multiple clinical phenotypes and biological endotypes that differ in their underlying inflammatory mechanisms, disease severity, treatment response, and long-term prognosis. Clinically, asthma can be classified into allergic, non-allergic, late-onset, obesity-associated, and severe phenotypes, whereas endotypes are broadly characterised by type 2 (T2)-high and T2-low inflammatory pathways [60]. Despite these well-recognised distinctions, most studies investigating cognition have treated asthma as a single disease entity rather than accounting for its biological and clinical heterogeneity.

Consequently, relatively few investigations have comprehensively characterised disease severity, asthma control, exacerbation frequency, lung function, inflammatory phenotype, or treatment exposure when examining cognitive outcomes. This lack of detailed phenotyping makes it difficult to determine whether cognitive impairment reflects asthma itself or the cumulative effects of more severe disease, chronic systemic inflammation, repeated exacerbations, or long-term treatment exposure.

Nevertheless, emerging evidence suggests that certain asthma characteristics may influence cognitive vulnerability. Longer disease duration and poorer lung function have been associated with reduced cognitive performance in adults with asthma [16], while poor asthma control has also been linked to worse cognitive function in older adults, although this relationship became non-significant after adjustment for confounding variables [15]. More recent evidence further suggests that systemic inflammation may partially mediate the association between asthma and cognitive dysfunction in older adults [61]. In addition, nationwide cohort data indicate that dementia risk among adults with asthma varies according to age, sex, and comorbidity burden, raising the possibility that susceptibility to cognitive decline differs across clinically distinct patient subgroups [40].

## 6. Clinical Consequences of Cognitive Impairment

### 6.1. Self-Management and Adherence

Cognitive impairment may compromise effective asthma self-management by disrupting the cognitive processes required to execute complex treatment-related behaviours, including attention, memory, planning, and procedural learning [48]. Successful asthma management depends not only on the availability of effective therapies but also on the patient’s ability to remember daily controller medication, recognise dosing schedules, interpret treatment instructions, and perform inhaler techniques accurately. Consequently, even subtle cognitive deficits may reduce adherence and increase the risk of poor disease control. In older adults with asthma, cognitive function has been shown to partly explain the relationship between health literacy and asthma medication use, including adherence to inhaled controller therapy and correct inhaler technique [62]. Similarly, Becker et al. reported that cognitive impairment is common among older adults with asthma and is associated with poorer self-management behaviours, highlighting cognition as an important but frequently overlooked determinant of treatment effectiveness [47].

Impaired inhaler technique represents an additional mechanism through which cognitive dysfunction may adversely influence asthma outcomes. Correct inhaler use requires patients to remember a sequence of device-specific steps, coordinate inhalation with actuation when appropriate, and retain these skills over time. However, poor inhaler technique remains one of the most common and persistent barriers to effective asthma management. Recent evidence indicates that inhaler technique frequently deteriorates following initial education, emphasising the importance of regular reassessment and reinforcement [63]. Although asthma-specific studies directly linking cognitive impairment with inhaler technique remain limited, evidence from chronic airway disease populations suggests that cognitive dysfunction independently predicts the ability to acquire and maintain correct inhaler technique after training [64]. Collectively, these findings suggest that cognitive impairment may indirectly contribute to poor asthma outcomes by reducing adherence and limiting the effective delivery of inhaled therapies.

### 6.2. Symptom Perception and Disease Control

Accurate symptom perception is fundamental to effective asthma self-management, enabling patients to recognise changes in disease activity, initiate appropriate treatment, and seek timely medical attention. Although self-management is a cornerstone of guideline-based asthma care and improves clinical outcomes, its effectiveness depends on a combination of cognitive, behavioural, and psychosocial factors. A recent systematic review identified multiple barriers to successful self-management, including medication-related concerns, inadequate education, comorbidities, psychological distress, limited healthcare access, and poor patient–healthcare professional partnerships, all of which may compromise disease control and treatment adherence [65]. Cognitive impairment may further exacerbate these challenges by disrupting attention, memory, and executive functioning, thereby reducing an individual’s ability to accurately interpret symptom severity and respond appropriately to clinical deterioration.

Consistent with this concept, poorer cognitive performance in older adults with asthma has been associated with reduced symptom perception and lower adherence to recommended self-management behaviours, suggesting that cognitive dysfunction may contribute to poorer disease control and adverse clinical outcomes [47]. Experimental evidence also provides biological support for this association. Chronic allergic airway inflammation has been shown to induce memory impairment and synaptic dysfunction in experimental models, supporting the biological plausibility that asthma-related inflammatory processes may contribute to cognitive dysfunction [66].

Importantly, symptom perception is increasingly recognised as a dynamic process rather than a fixed individual characteristic. Perception of respiratory symptoms is influenced by cognitive and affective factors, including attention, expectations, emotional state, and previous symptom experiences [13]. Consequently, disturbances in cognition may impair accurate symptom appraisal, while coexisting psychological conditions may further amplify these effects. Indeed, depression and cognitive impairment in older adults with asthma have been associated with altered symptom perception, impaired self-management, poorer disease control, more frequent exacerbations, and increased healthcare utilisation [67]. However, whether cognitive impairment independently increases the risk of severe exacerbations or asthma-related mortality remains unknown because these outcomes have not been specifically evaluated in prospective studies.

Delayed healthcare-seeking may represent another clinically important consequence of cognitive dysfunction. Deficits in memory, information processing, and decision-making may impair an individual’s ability to recognise worsening symptoms and seek appropriate medical attention before significant clinical deterioration occurs. Although direct evidence linking cognitive impairment to delayed healthcare utilisation in asthma remains limited, observational studies have consistently demonstrated associations between impaired cognition, poorer self-management, and suboptimal disease control in older adults with asthma [68]. This hypothesis is further supported by evidence from chronic respiratory disease, where cognitive impairment has been proposed as an intermediary mechanism linking disease burden with impaired symptom appraisal, reduced self-management, increased healthcare utilisation, and poorer clinical outcomes [48]. While direct evidence in asthma remains limited, these findings suggest that similar mechanisms are likely to contribute to adverse disease outcomes in susceptible patients.

### 6.3. Functional Outcomes

Cognitive impairment may have important functional consequences across the lifespan by affecting educational achievement during childhood and occupational performance in adulthood. Although asthma-related functional impairment is multifactorial, cognitive dysfunction may represent an important mechanism through which disease burden translates into reduced academic and work productivity.

#### 6.3.1. Academic Performance

Asthma may adversely affect academic performance through the combined effects of cognitive dysfunction, disease burden, and psychosocial factors. Successful learning relies on sustained attention, working memory, executive function, and efficient information processing. Consequently, the cognitive deficits reported in some children with asthma may interfere with classroom engagement, learning, memory consolidation, and examination performance. These effects may be further compounded by nocturnal symptoms, sleep fragmentation, daytime fatigue, recurrent exacerbations, psychological distress, and treatment-related disruption [69]. Frequent school absence represents an additional pathway through which asthma may impair educational attainment, as healthcare visits, hospitalisations, and recovery periods reduce instructional time and interrupt learning continuity.

Current evidence suggests that educational outcomes are influenced more by disease control and overall morbidity than by the diagnosis of asthma itself. In a large Swedish population-based study involving more than 570,000 children, uncontrolled asthma was associated with lower grade-point averages, poorer national examination performance, and a greater likelihood of ineligibility for upper-secondary education. Although most associations were attenuated following sibling comparisons, indicating important contributions from familial and socioeconomic factors, the association between contemporaneous uncontrolled asthma and poorer school performance remained significant, supporting an independent disease-related effect [70]. Similarly, longitudinal studies have linked paediatric asthma with poorer performance on standardised academic assessments over several school years, while recent evidence continues to identify asthma-related absenteeism, disturbed sleep, emergency healthcare utilisation, and socioeconomic disadvantage as important contributors to educational disruption [71].

These findings indicate that reduced academic performance is unlikely to result from cognitive impairment in isolation. Rather, educational outcomes probably reflect the interaction between impaired attention or memory, poor asthma control, sleep disturbance, school absenteeism, anxiety, environmental exposures, parental support, and access to effective healthcare. This multifactorial nature may explain why some studies report only modest differences between children with and without asthma, whereas more consistent adverse educational outcomes are observed among those with uncontrolled, persistent, or severe disease [72]. Although direct studies examining cognition as a mediator remain limited, the available evidence suggests that optimising asthma control, reducing nocturnal symptoms and exacerbations, and minimising school absence may help preserve educational participation and academic achievement.

#### 6.3.2. Occupational Performance

In adulthood, cognitive impairment may contribute to reduced occupational functioning by amplifying the effects of respiratory symptoms and chronic disease burden on work performance. Asthma affects productivity through both absenteeism, defined as time missed from work, and presenteeism, whereby individuals remain at work but perform below their usual capacity. Respiratory symptoms, fatigue, sleep disruption, psychological distress, recurrent healthcare utilisation, and physical limitations may directly impair work capacity. Superimposed cognitive dysfunction involving attention, memory, planning, and executive function may further reduce concentration, decision-making, task completion, and the ability to perform complex or safety-critical occupational activities. In addition, cognitive impairment may indirectly reduce productivity by compromising medication adherence, inhaler technique, symptom recognition, and overall disease control, thereby increasing the likelihood of exacerbations and work disruption.

Recent evidence demonstrates substantial occupational impairment among adults with asthma. A 2024 population-based study found that workers with both work-related and non-work-related asthma experienced significantly poorer overall work productivity than workers without asthma, with the greatest impairment observed among those with work-related disease. Work-related asthma was associated with markedly higher levels of absenteeism and presenteeism, increased healthcare utilisation, and a greater likelihood of income reduction attributable to respiratory symptoms [73]. Similarly, studies of severe asthma consistently demonstrate that poor disease control is associated with greater work impairment, whereas improved symptom control and biologic therapies are associated with meaningful improvements in work productivity and functional participation [74,75]. A multinational study further estimated that asthma was associated with an overall productivity loss of approximately 36%, although the magnitude varied across healthcare systems and occupational settings [76]. Collectively, these findings suggest that cognitive dysfunction may represent one of several interacting mechanisms linking asthma severity with impaired occupational performance and reduced socioeconomic participation.

### 6.4. Quality of Life

Health-related quality of life (HRQoL) represents one of the most important patient-centred outcomes in asthma and is influenced by the combined effects of physical symptoms, psychological wellbeing, social participation, and functional capacity. Cognitive impairment may further increase this burden by compromising self-management, symptom appraisal, independence, and daily functioning, thereby adversely affecting overall HRQoL [77].

Several clinical factors have consistently been associated with poorer HRQoL in asthma. A large multicentre study identified advanced age, lower educational attainment, poor asthma control, greater disease severity, and previous hospitalisation as independent predictors of reduced HRQoL among adults with asthma [78]. The burden is particularly evident in severe asthma, where persistent symptoms, recurrent exacerbations, multiple comorbidities, and intensive treatment requirements collectively impair physical, psychological, social, and occupational functioning [79]. Although the direct contribution of cognitive impairment to HRQoL has not been comprehensively investigated, it is biologically and clinically plausible that cognitive dysfunction further amplifies these adverse effects by reducing an individual’s ability to manage disease effectively and maintain independence in everyday life.

The relationship between cognitive dysfunction and psychological wellbeing is likely to be bidirectional. Cognitive deficits may reduce an individual’s ability to cope with the demands of chronic disease, thereby increasing emotional distress, while anxiety and depression may further accelerate cognitive decline through behavioural and neurobiological mechanisms. Consistent with this concept, patients with asthma have been shown to exhibit significantly higher levels of anxiety, depressive symptoms, and cognitive dysfunction than healthy controls, with these neuropsychological abnormalities correlating with tobacco smoke exposure and the severity of airflow obstruction [80].

Evidence from broader chronic disease populations further supports this relationship. Cognitive impairment has been independently associated with poorer quality of life among older adults with chronic illnesses, with depressive symptoms and frailty partially mediating this association [81]. Although these findings are not specific to asthma, they provide a plausible framework through which psychological distress may amplify the impact of cognitive dysfunction on HRQoL. This concept is further supported by a recent Saudi Arabian study demonstrating that asthma was independently associated with mild cognitive impairment as well as symptoms of anxiety and depression, while poorly controlled asthma further increased the likelihood of anxiety, and older adults with asthma exhibited a substantially greater risk of mild cognitive impairment [36]. Taken together, these findings suggest that cognitive dysfunction, psychological distress, and poor asthma control interact to produce a cumulative reduction in health-related quality of life, reinforcing the need for a multidisciplinary approach that addresses both respiratory and neuropsychological health.

## 7. Potential Mechanisms

The biological mechanisms linking asthma to cognitive impairment remain incompletely understood and are likely to be multifactorial. Asthma is characterised by chronic airway inflammation, recurrent airflow limitation, and varying degrees of systemic immune activation, all of which may influence brain structure and function through interconnected biological pathways. Several mechanisms have been proposed, including intermittent hypoxia during exacerbations, chronic systemic and neuroinflammation, oxidative stress, vascular dysfunction, sleep disturbance, psychological comorbidities, and corticosteroid exposure. Rather than acting independently, these mechanisms are likely to interact and reinforce one another, promoting neuronal injury, synaptic dysfunction, impaired neuroplasticity, and ultimately cognitive decline. Although experimental and epidemiological studies provide strong biological plausibility for these pathways, direct mechanistic evidence in patients with asthma remains limited. Consequently, well-designed translational and longitudinal studies are needed to define the relative contribution of individual mechanisms and determine whether they directly mediate the relationship between asthma and long-term cognitive impairment.

### 7.1. Intermittent Hypoxia During Exacerbations

**Figure 2:**
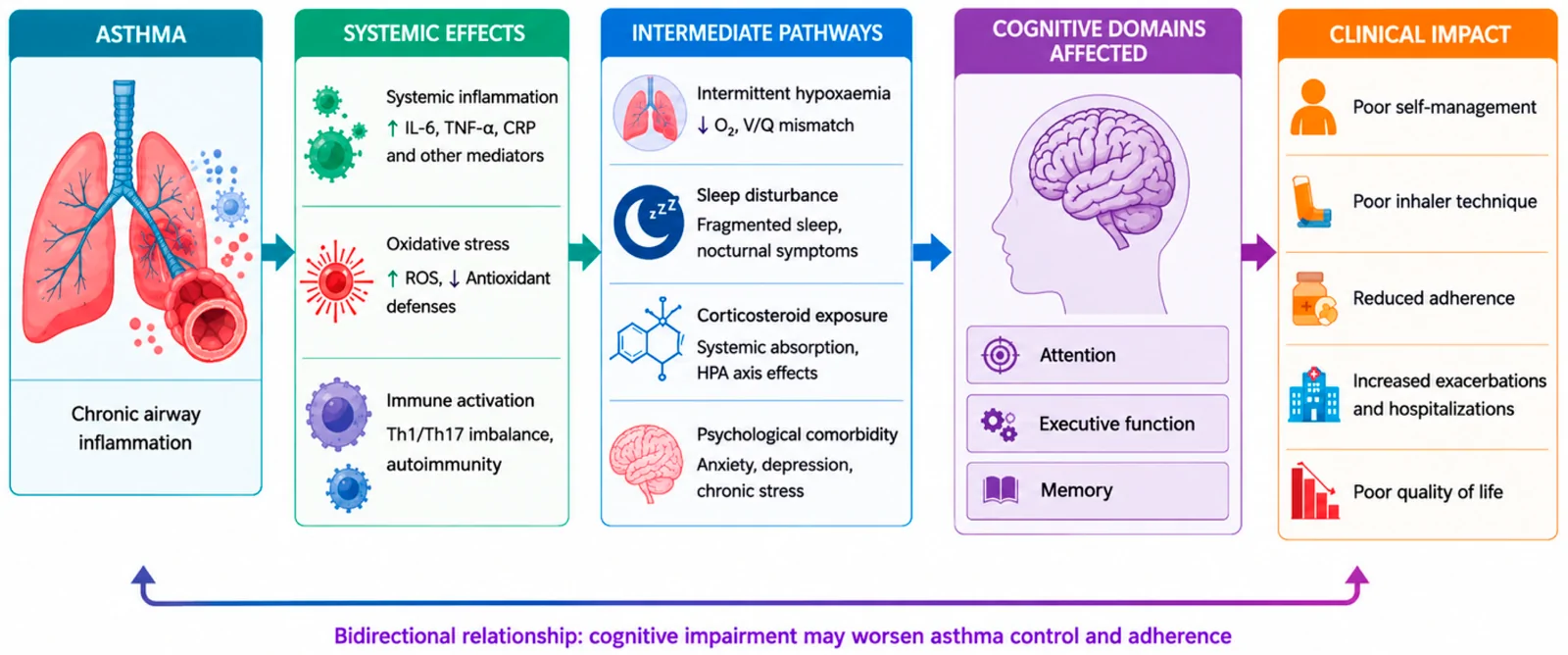
Mechanisms linking asthma to cognitive impairment. Arrows indicate the proposed pathway linking asthma, systemic effects, intermediate mechanisms, cognitive impairment, and subsequent clinical consequences. The bidirectional arrow indicates that cognitive impairment may, in turn, negatively affect asthma control and treatment adherence. The figure was created with the assistance of ChatGPT (OpenAI; https://chatgpt.com) for visual presentation.

As illustrated in Figure 2, intermittent hypoxia represents one of several interconnected biological mechanisms through which asthma may contribute to cognitive impairment. During acute asthma exacerbations, worsening airflow obstruction, ventilation–perfusion mismatch, and increased respiratory workload may result in transient arterial oxygen desaturation, particularly during severe or life-threatening episodes [82]. Repeated exacerbations may therefore expose the brain to recurrent cycles of hypoxia–reoxygenation, especially in children, older adults, and individuals with pre-existing cerebrovascular or neurodegenerative vulnerability [83]. Although direct clinical evidence remains limited, intermittent hypoxia provides a biologically plausible pathway linking pulmonary dysfunction with neuronal injury and cognitive decline.

Experimental studies suggest that recurrent hypoxia may initiate a complex cascade of systemic and cellular responses (Figure 2). Cycles of hypoxia–reoxygenation may promote excessive generation of reactive oxygen species (ROS), mitochondrial dysfunction, and impaired antioxidant defence mechanisms, creating a state of persistent oxidative stress. These changes may activate multiple redox-sensitive signalling pathways, including NF-κB, MAPK, PI3K/Akt, and JAK/STAT, which regulate inflammatory responses, apoptosis, and cellular survival [84,85,86]. In parallel, intermittent hypoxia has been suggested to enhance inflammatory signalling through activation of hypoxia-inducible factor-1α (HIF-1a)-dependent pathways, particularly the HIF-1a /NLRP3/IL-1β inflammasome, leading to increased production of pro-inflammatory cytokines including IL-1β, IL-6, and TNF-a [87]. Rather than representing separate biological processes, oxidative stress and inflammation appear to amplify one another, generating a self-perpetuating cycle of cellular injury. However, much of the evidence supporting these pathways is derived from experimental models or other disease context, and their direct relevance to cognitive impairment in human asthma remain to be established.

These peripheral inflammatory and oxidative responses may subsequently affect the central nervous system through multiple interconnected mechanisms. Increased circulating inflammatory mediators, together with oxidative endothelial injury, may impair cerebrovascular integrity, increase blood–brain barrier permeability, activate resident microglia, and promote chronic neuroinflammation [88,89,90]. Persistent microglial activation may further amplify oxidative stress and mitochondrial dysfunction, leading to synaptic injury, impaired neuronal connectivity, and reduced neuroplasticity. These changes are accompanied by reduced expression of neurotrophic factors, particularly brain-derived neurotrophic factor (BDNF), which plays a central role in neuronal survival, synaptic plasticity, and memory formation [84,88,91]. Brain regions with high metabolic demand, including the hippocampus and prefrontal cortex, appear especially susceptible to hypoxic injury, potentially resulting in impairments in attention, executive function, memory, and processing speed [92].

Despite this compelling biological rationale, direct clinical evidence supporting intermittent hypoxia as an independent determinant of cognitive impairment in asthma remains limited. Earlier studies demonstrated that lower arterial oxygen saturation in older adults with asthma was associated with selective cognitive deficits [93]. More recently, longitudinal data have shown that children with asthma, particularly those with earlier disease onset, exhibit slower improvement in episodic memory together with poorer attention, inhibitory control, and processing speed than healthy peers [42]. However, neither study directly quantified exacerbation-related hypoxaemia or cumulative hypoxic burden, making it difficult to determine whether intermittent hypoxia independently explains these cognitive abnormalities.

Importantly, intermittent hypoxia should not be considered in isolation. During asthma exacerbations, hypoxaemia frequently occurs alongside systemic inflammation, oxidative stress, sleep disruption, psychological distress, and increased exposure to systemic corticosteroids (Figure 2). These mechanisms are likely to interact synergistically rather than independently, collectively increasing neuronal vulnerability and accelerating cognitive dysfunction. Accordingly, cognitive impairment in asthma is unlikely to arise from intermittent hypoxia alone but instead reflects the cumulative effects of multiple interacting biological insults operating throughout the course of the disease.

### 7.2. Chronic Inflammation and Systemic Effects

Chronic systemic inflammation represents another biologically plausible mechanism linking asthma with cognitive impairment. Although airway inflammation is the hallmark of asthma, systemic inflammatory responses are particularly evident in severe or difficult-to-treat asthma, recurrent exacerbations, obesity-associated asthma, and type 2-low inflammatory phenotypes, although elevated circulating inflammatory markers have also been described in type 2-high disease. This persistent inflammatory burden may contribute to extrapulmonary complications through ongoing immune activation, oxidative stress, endothelial dysfunction, and metabolic dysregulation [94]. Supporting this hypothesis, a recent NHANES study of older adults demonstrated that asthma was independently associated with poorer cognitive performance, with the systemic immune-inflammation index (SII) partially mediating this relationship, suggesting that chronic systemic inflammation may contribute to cognitive vulnerability in asthma [61].

Peripheral inflammatory signals may influence the central nervous system through several interconnected pathways. Circulating cytokines and other immune mediators can activate cerebral endothelial cells, stimulate neural afferent pathways, and increase blood–brain barrier permeability, thereby facilitating communication between the peripheral immune system and the brain. Once activated, microglia may sustain neuroinflammation through the release of pro-inflammatory cytokines, including IL-1β, IL-6, and TNF-a, promoting synaptic dysfunction, impaired neuroplasticity, and neuronal injury. Although these mechanisms are well established in neurodegenerative disorders, their contribution to asthma-related cognitive impairment remains largely hypothetical because direct evidence in asthma is limited [95].

The neurological consequences of systemic inflammation may also differ according to asthma phenotype. Type 2-high asthma is predominantly characterised by eosinophilic inflammation driven by IL-4, IL-5, IL-13, and epithelial alarmins, whereas type 2-low asthma is more frequently associated with neutrophilic inflammation involving IL-1β, IL-6, IL-17, and TNF-a, often accompanied by obesity, metabolic dysfunction, and cardiovascular disease, all of which are independently associated with cognitive impairment [96,97]. Despite these biological differences, few clinical studies have examined cognitive outcomes according to inflammatory phenotype. Consequently, whether specific asthma phenotypes confer greater neurological vulnerability remains unknown. Future studies integrating inflammatory biomarkers, detailed phenotyping, and longitudinal cognitive assessment will be essential to clarify these relationships.

### 7.3. Sleep Disturbance (Nocturnal Asthma)

Sleep disturbance represents another important pathway through which asthma may influence cognitive function. Individuals with asthma commonly experience difficulty initiating and maintaining sleep, sleep fragmentation, early morning awakening, and excessive daytime sleepiness, particularly when asthma is poorly controlled [98]. Persistent disruption of normal sleep architecture may impair attention, memory consolidation, processing speed, and executive function through a combination of daytime somnolence, circadian dysregulation, oxidative stress, and neuroinflammatory activation [99].

Asthma-specific evidence increasingly supports this association. A recent multicentre study published in 2026 reported that poor sleep quality was strongly associated with cognitive impairment among adults with asthma, although its cross-sectional design precluded conclusions regarding causality [55]. Similarly, experimental studies in children with asthma have demonstrated that sleep disruption adversely affects daytime attention and alertness, supporting a potential mechanistic link between nocturnal asthma symptoms and impaired cognitive performance [100].

Sleep-related comorbidities may further amplify this risk. Obstructive sleep apnoea is common among individuals with asthma and is characterised by recurrent arousals, intermittent hypoxaemia, systemic inflammation, and endothelial dysfunction, all recognised contributors to neurocognitive impairment [101]. Importantly, the relationship between asthma and sleep disturbance appears to be bidirectional. Nocturnal symptoms impair sleep quality, whereas inadequate sleep may increase systemic inflammation, alter symptom perception, reduce treatment adherence, and increase susceptibility to exacerbations. Consistent with this concept, a large prospective cohort study identified poor sleep quality as an independent predictor of incident asthma, suggesting that sleep disturbance may actively contribute to asthma pathogenesis rather than simply arise as a consequence of established disease [102].

### 7.4. Medication Effects

Corticosteroid exposure has been proposed as an additional mechanism linking asthma with cognitive dysfunction, although the available evidence remains inconclusive. Systemic corticosteroids readily cross the blood–brain barrier and activate glucocorticoid receptors that are highly expressed within the hippocampus, a brain region critically involved in learning and memory. Prolonged or repeated exposure may disrupt hypothalamic–pituitary–adrenal axis regulation, reduce neurogenesis, alter synaptic plasticity, and increase oxidative stress, potentially contributing to hippocampal-dependent cognitive dysfunction [103,104].

Evidence supporting these effects is emerging but remains incomplete. A recent systematic review reported that glucocorticoid exposure was associated with impaired cognition, particularly deficits involving hippocampal-dependent memory, although cognitive adverse effects were rarely assessed systematically in randomised clinical trials [105]. Similarly, a recent systematic review and meta-analysis demonstrated that glucocorticoid therapy is associated with a substantial burden of neuropsychiatric adverse effects, particularly depression, mania, and delirium [106].

The neurological effects of inhaled corticosteroids (ICS) are considerably less certain because systemic exposure is substantially lower than with oral or intravenous corticosteroids. Nevertheless, prolonged ICS therapy may produce systemic effects, including hypothalamic–pituitary–adrenal axis suppression, reduced bone mineral density, ocular complications, and immune dysfunction, although direct evidence linking ICS to cognitive impairment remains sparse [107]. A 2024 observational study involving 139 individuals with asthma or COPD reported a higher prevalence of cognitive impairment among patients receiving inhaled corticosteroids for more than two years; however, the mixed disease population, cross-sectional design, and inadequate adjustment for disease severity substantially limit interpretation [108].

Importantly, corticosteroid exposure should be interpreted within the broader clinical context of asthma severity. Inhaled corticosteroids remain the cornerstone of asthma management and reduce exacerbations, hypoxaemia, sleep disruption, and the need for systemic corticosteroids, all of which may indirectly protect cognitive function by reducing cumulative disease burden. Furthermore, medication exposure is closely linked to disease severity, creating substantial confounding by indication, whereby patients receiving higher corticosteroid doses often have more severe asthma, greater systemic inflammation, and more frequent exacerbations that may themselves contribute to cognitive impairment [109,110,111]. Accordingly, corticosteroid therapy should currently be regarded as a potential modifier of cognitive outcomes rather than an established independent cause.

### 7.5. Interaction with Mental Health Conditions

Psychological comorbidities, particularly anxiety and depression, may represent an important intermediary between asthma and cognitive impairment. Both conditions are independently associated with deficits in attention, memory, executive function, and processing speed, making it difficult to distinguish cognitive changes attributable directly to asthma from those related to coexisting psychiatric illness. At the same time, poor asthma control, recurrent exacerbations, nocturnal symptoms, and treatment burden may increase psychological distress, whereas anxiety and depression may impair symptom perception, medication adherence, and self-management, creating a bidirectional cycle that may further worsen cognitive and clinical outcomes [36,80,112].

Growing evidence indicates that the relationship between asthma and mental health is itself bidirectional. Shared inflammatory, psychoneuroimmunological, and psychosocial pathways—including chronic airway inflammation, altered neuroimmune signalling, and dysregulation of stress-response systems—may contribute to both respiratory disease and psychiatric disorders [113]. Population-based studies consistently demonstrate a higher prevalence of anxiety disorders and affective illnesses, particularly panic disorder, generalised anxiety disorder, and specific phobias, among individuals with asthma, supporting a robust association between asthma and psychological morbidity [114].

The clinical consequences of this interaction extend beyond mental health alone. A recent systematic review identified anxiety and depression as major determinants of reduced quality of life in asthma, acting alongside physical disease burden to impair self-management, daily functioning, and overall clinical outcomes [115]. Because anxiety and depression independently affect multiple cognitive domains, psychiatric comorbidity may substantially contribute to the cognitive impairment observed in asthma.

## 8. Special Populations

The relationship between asthma and cognitive impairment is unlikely to be uniform across the lifespan or across levels of disease severity. Children and adolescents may be particularly vulnerable because asthma develops during critical stages of brain maturation, whereas individuals with severe asthma experience greater cumulative exposure to systemic inflammation, recurrent exacerbations, intermittent hypoxaemia, sleep disruption, and corticosteroid therapy. In older adults, these asthma-related factors interact with age-related neurodegenerative, vascular, and metabolic changes, making both the origin and clinical consequences of cognitive impairment more complex.

### 8.1. Children and Adolescents

Childhood and adolescence represent periods of rapid neurodevelopment during which the hippocampus, prefrontal cortex, and associated neural networks undergo substantial structural and functional maturation. These developmental processes underpin the acquisition of memory, attention, executive function, and processing speed. Consequently, asthma occurring during these sensitive developmental periods may have effects that extend beyond respiratory health by altering normal neurodevelopmental trajectories.

A recent review by Christopher-Hayes and Ghetti proposed that persistent airway and systemic inflammation, recurrent intermittent hypoxaemia during exacerbations, sleep disturbance, corticosteroid exposure, psychosocial stress, and environmental factors such as air pollution collectively interact to influence brain development, particularly among children with early-onset or poorly controlled asthma [38]. Rather than producing only transient cognitive deficits, prolonged exposure to these factors during critical periods of brain maturation may alter long-term cognitive trajectories.

Clinical evidence supports this biological hypothesis. In the Childhood Asthma Management Program (CAMP), Annett et al. demonstrated that children with mild-to-moderate asthma had overall intellectual abilities comparable with normative expectations but exhibited subtle impairments in sustained attention, executive function, and memory, suggesting that asthma selectively affects higher-order cognitive processes rather than global intellectual ability [116]. These observations are reinforced by longitudinal findings from the Adolescent Brain Cognitive Development (ABCD) Study, in which children with asthma demonstrated poorer episodic memory and executive function than matched controls. Moreover, earlier disease onset was associated with slower improvement in episodic memory over time, supporting the hypothesis that prolonged exposure to asthma during childhood may adversely influence normal neurocognitive development [42].

These observations highlight the potential importance of achieving optimal asthma control early in life. Effective disease management may reduce cumulative exposure to several mechanisms implicated in altered neurodevelopment, including recurrent exacerbations, nocturnal symptoms, sleep fragmentation, and intermittent hypoxaemia. Although direct evidence demonstrating improvements in cognitive outcomes remains unavailable, a recent meta-analysis reported that community-based asthma interventions significantly reduced emergency department attendance, hospitalisations, daytime and nocturnal symptoms, and bronchodilator use while improving adherence to asthma action plans [117]. Collectively, these findings emphasise the importance of sustained asthma control during periods of rapid brain development.

Maintaining disease control remains equally important during adolescence, a developmental stage characterised by continuing physiological, psychological, and neurocognitive maturation. Behavioural changes, increasing independence, treatment non-adherence, and the emergence of severe or difficult-to-treat asthma may increase cumulative exposure to factors capable of influencing long-term cognitive health [118].

The functional consequences of these neurodevelopmental changes may become apparent through educational and behavioural outcomes. Although studies evaluating academic achievement have produced mixed results, differences in asthma severity, disease control, and socioeconomic context probably account for much of this variability. Nevertheless, evidence consistently demonstrates that poor asthma control is associated with increased school absenteeism. A large population-based study further showed that asthma-related absenteeism was associated with greater emergency healthcare utilisation together with modifiable environmental and socioeconomic barriers, highlighting the broader educational consequences of inadequately controlled disease [119].

More recent longitudinal evidence further suggests that asthma, particularly when associated with greater healthcare utilisation or medication requirements, is linked to poorer performance on standardised academic assessments [120]. Similarly, an urban paediatric cohort demonstrated that asthma-related morbidity, sleep disruption, and school absence were associated with less favourable educational outcomes, although socioeconomic disadvantage and environmental exposures also contributed substantially [72]. A multicentre study likewise reported that inadequate asthma control was associated with greater school absenteeism, lower academic achievement, and poorer school-related quality of life [121]. Taken together, these findings suggest that educational attainment is influenced less by the diagnosis of asthma itself than by the cumulative effects of poor disease control, recurrent exacerbations, sleep disturbance, healthcare utilisation, and social disadvantage.

Behavioural and emotional difficulties may arise through similarly complex biological and psychosocial pathways. A systematic review concluded that psychosocial factors influence asthma through neuroendocrine, immunological, behavioural, and disease-management mechanisms, while behavioural interventions—including self-management education, breathing training, and exercise programmes—can improve asthma outcomes within an integrated biopsychosocial framework [122]. Complementing these findings, an integrative review reported that asthma continues to impose substantial physical, psychological, and social burdens on children and adolescents who, despite demonstrating willingness and capacity to develop self-management skills, frequently have unmet support needs [123].

Children with asthma also experience higher rates of anxiety, depression, attentional symptoms, and internalising behaviours than their healthy peers, each of which may independently impair classroom engagement, learning, and cognitive performance. Conversely, academic difficulties, activity restrictions, and perceived differences from peers may themselves increase psychological distress, creating a bidirectional interaction between emotional wellbeing, disease burden, and cognitive functioning.

### 8.2. Severe Asthma Patients

Severe asthma represents the most clinically complex end of the asthma spectrum and is characterised by persistent symptoms, recurrent exacerbations, and a substantial treatment burden despite optimised therapy. Although only a small proportion of individuals with asthma develop severe disease, this population accounts for a disproportionate share of hospitalisations, healthcare utilisation, corticosteroid exposure, and impaired quality of life. Consequently, patients with severe asthma are exposed to multiple biological and clinical factors that have independently been associated with cognitive dysfunction, including chronic systemic inflammation, recurrent intermittent hypoxaemia, sleep disruption, psychological distress, physical inactivity, and multimorbidity. Collectively, these cumulative disease-related insults may increase vulnerability to neurocognitive impairment beyond the effects of airway disease alone [94,124].

From a biological perspective, severe asthma is increasingly recognised as a systemic inflammatory disorder extending beyond the airways. Persistent immune activation, oxidative stress, endothelial dysfunction, and metabolic dysregulation may contribute to cerebrovascular injury, blood–brain barrier dysfunction, microglial activation, and impaired synaptic plasticity. Recurrent exacerbations may further amplify these processes through repeated inflammatory surges and intermittent hypoxaemia, providing a biologically plausible explanation for the increased neurocognitive vulnerability observed in severe asthma. Nevertheless, direct studies linking inflammatory phenotypes of severe asthma with objective neuropsychological outcomes remain scarce, and causality has yet to be established [18,94,125].

Cognitive impairment may also have important practical implications for disease management. Effective treatment of severe asthma requires patients to interpret symptoms accurately, adhere to complex medication regimens, use inhaler devices correctly, monitor disease control, implement personalised action plans, and seek timely medical attention during deterioration. Even subtle deficits in memory, attention, executive function, or health literacy may compromise these tasks, leading to poorer adherence, delayed recognition of worsening symptoms, and increased exacerbation risk. This creates a potentially self-perpetuating cycle in which uncontrolled disease further increases the burden of factors associated with cognitive dysfunction. Emerging evidence suggests that cognitive impairment may be relatively common among individuals with severe asthma, highlighting cognition as a potentially important but under-recognised component of comprehensive severe asthma management [126,127].

Patients with severe asthma are substantially more likely to require repeated courses of systemic corticosteroids or long-term oral corticosteroid therapy despite recent advances in biologic treatments. Although systemic corticosteroids remain highly effective for controlling acute airway inflammation, cumulative exposure is increasingly recognised as an important contributor to treatment-related morbidity. Beyond their well-established metabolic, cardiovascular, skeletal, infectious, and endocrine adverse effects, prolonged glucocorticoid exposure may influence cognitive function through disruption of hypothalamic–pituitary–adrenal axis regulation, impaired hippocampal neuroplasticity, and altered glucocorticoid receptor signalling. Because glucocorticoid signalling plays an essential role in learning, memory, and stress adaptation, excessive or prolonged exposure may increase neurocognitive vulnerability. However, direct evidence in asthma remains limited, and disentangling the effects of corticosteroid exposure from those of severe disease remains challenging because both are closely linked [128,129,130,131].

### 8.3. Older Adults

Older adults may represent the population most vulnerable to asthma-related cognitive impairment because disease-specific factors occur in the context of age-related reductions in cognitive reserve and increasing multimorbidity. Ageing is accompanied by progressive changes in memory, attention, executive function, and processing speed, together with a higher prevalence of cardiovascular disease, diabetes, obesity, obstructive sleep apnoea, depression, and polypharmacy, all of which independently increase the risk of cognitive decline [132,133]. Consequently, asthma may amplify existing age-related neurocognitive vulnerability rather than act as an isolated cause of cognitive impairment.

Emerging evidence supports an association between asthma and poorer cognitive performance in later life, although findings remain inconsistent. A 2024 analysis of older adults from the NHANES cohort found that asthma was associated with lower processing speed and attention, with systemic inflammation partially mediating this relationship [61]. Furthermore, depression and cognitive impairment are frequently under-recognised and inadequately addressed in the clinical management of older adults with asthma, despite being common comorbidities in this population [67]. Cognitive impairment has important implications for asthma self-management, as successful disease control depends on correct inhaler technique, adherence to maintenance therapy, recognition of worsening symptoms, and appropriate implementation of asthma action plans. Deficits in memory, attention, and executive function may compromise these tasks, contributing to poor asthma control, recurrent exacerbations, and increased healthcare utilisation. Collectively, these findings suggest that cognitive impairment may represent an important but frequently under-recognised determinant of asthma control in older adults, reinforcing the need for routine cognitive assessment in selected high-risk patients [44,134].

## 9. Limitations of Current Evidence

### 9.1. Heterogeneity in Study Design and Populations

Interpretation of the current evidence remains limited by substantial methodological heterogeneity. Studies differ considerably in design, participant characteristics, asthma definitions, disease severity, treatment exposure, cognitive outcomes, and duration of follow-up. Furthermore, asthma has frequently been analysed as a single disease entity despite its recognised clinical and biological heterogeneity, while important comorbidities—including obesity, cardiovascular disease, depression, anxiety, obstructive sleep apnoea, and smoking history—have not been consistently accounted for. Consequently, distinguishing true biological differences from methodological variation remains challenging [18,38].

### 9.2. Lack of Standardised Cognitive Assessment Tools

Interpretation of the current literature is further complicated by the lack of standardisation in cognitive assessment. Studies have employed diverse screening instruments and neuropsychological tests that differ in their sensitivity, specificity, and the cognitive domains assessed, while cognitive impairment has been defined using inconsistent diagnostic thresholds and outcome measures. Consequently, differences in reported prevalence and cognitive deficits may reflect methodological variation rather than genuine differences in cognitive function. Moreover, the use of brief screening tools in many studies may underestimate subtle impairments, particularly in executive function and processing speed, which appear to be among the domains most consistently affected in asthma. The absence of a standardised cognitive assessment framework therefore remains a major barrier to comparing findings across studies and accurately defining the cognitive profile associated with asthma. Establishing a standardised cognitive assessment framework that combines validated screening instruments with domain-specific neuropsychological testing should therefore represent a priority for future research. Such an approach would facilitate comparison across studies, improve phenotypic characterisation, and provide a more accurate understanding of the cognitive profile associated with asthma [52,53].

### 9.3. Predominance of Cross-Sectional Studies

The predominance of cross-sectional study designs represents another major limitation of the current evidence. Although these studies consistently demonstrate an association between asthma and poorer cognitive performance, they provide only a single snapshot of cognition and cannot establish the temporal sequence of events. Consequently, it remains unclear whether asthma contributes to progressive cognitive decline, whether cognitive impairment compromises asthma self-management and disease control, or whether both conditions arise from shared inflammatory, vascular, or behavioural mechanisms.

Although several longitudinal cohort studies have reported an increased risk of cognitive decline and dementia among individuals with asthma, prospective evidence remains limited and is characterised by considerable methodological variability, including differences in follow-up duration, outcome definitions, and adjustment for important confounding factors. Future longitudinal studies should combine serial neuropsychological assessment with detailed measures of asthma severity, disease control, inflammatory biomarkers, neuroimaging, and objective assessment of sleep and oxygenation to better define the temporal relationship between asthma and cognitive decline [18,37,40].

### 9.4. Limited Causal Inference

Despite accumulating evidence supporting an association between asthma and cognitive impairment, causality remains unproven. Asthma is a complex and heterogeneous disorder in which multiple factors associated with cognitive dysfunction—including systemic inflammation, intermittent hypoxaemia, sleep disturbance, corticosteroid exposure, psychiatric comorbidity, obesity, cardiovascular disease, and ageing—frequently coexist and interact. Consequently, distinguishing the direct effects of asthma from those attributable to cumulative disease burden or associated comorbidities remains challenging.

The absence of mechanistic studies directly linking asthma-related biological processes with objective cognitive outcomes, together with inconsistent adjustment for confounding variables, further limits causal inference. Establishing causality will require multidimensional prospective studies integrating detailed asthma phenotyping with serial neuropsychological assessment, neuroimaging, inflammatory and neurodegenerative biomarkers, and objective measures of sleep and oxygenation to define the relative contribution of individual pathogenic pathways.

## 10. Future Directions

### 10.1. Need for Longitudinal Cohort Studies

Future research should prioritise large, well-phenotyped longitudinal cohorts to determine whether asthma contributes to progressive cognitive decline across the lifespan. Integrating serial neuropsychological assessment with detailed asthma phenotyping, inflammatory biomarkers, neuroimaging, and objective measures of sleep and oxygenation will be essential to establish temporal relationships, identify susceptible patient populations, and clarify the biological mechanisms linking asthma with cognitive dysfunction.

### 10.2. Standardisation of Cognitive Outcome Measures

Progress in this field will also depend on greater methodological standardisation. The use of diverse screening instruments, assessment batteries, cognitive domains, and diagnostic thresholds currently limits comparison across studies and complicates evidence synthesis. Developing consensus recommendations for cognitive assessment in asthma research should therefore represent a research priority.

Rather than relying solely on global cognitive screening tools, future studies should incorporate validated, domain-specific neuropsychological batteries capable of detecting subtle impairments in attention, executive function, processing speed, and memory. Standardised reporting of cognitive outcomes, together with consistent adjustment for major confounding variables, would facilitate pooled analyses and improve characterisation of the cognitive phenotype associated with asthma.

### 10.3. Integration of Cognition into Asthma Management Research

Cognition should increasingly be recognised as a clinically relevant component of asthma management rather than merely an exploratory research outcome. Future observational studies and clinical trials should investigate whether cognitive impairment independently influences medication adherence, inhaler technique, symptom perception, exacerbation frequency, healthcare utilisation, and health-related quality of life.

Incorporating brief cognitive assessment into studies of severe asthma, biologic therapies, and corticosteroid-sparing strategies may also help determine whether improvements in disease control are accompanied by preservation or recovery of cognitive function. Such work could identify patients who would benefit from tailored education, simplified treatment regimens, or multidisciplinary support, thereby extending asthma management beyond traditional respiratory outcomes.

### 10.4. Potential Role of Biologic Therapies

The increasing use of targeted biologic therapies in severe asthma raises the possibility that the effective suppression of type 2 (T2) inflammation may influence cognitive outcomes, although this remains largely unexplored. Currently available biologics target distinct inflammatory pathways, including IgE (omalizumab), IL-5 or its receptors (mepolizumab, reslizumab, and benralizumab) IL-4Ra (dupilumab) and thymic stromal lymphopoietin (TSLP; Tezepelumab). These therapies have demonstrated substantial benefits in reducing agents, particularly mepolizumab, benralizumab, and dupilumab, have demonstrated oral corticosteroid-sparing effect in patient with severe asthma [96,135].

These clinical benefits provide a plausible pathway through which biologic therapy could indirectly influence cognitive health. By reducing inflammatory activity, exacerbation burden, and dependence on systemic corticosteroids, biologic therapies may potentially attenuate several factors implicated in asthma-associated cognitive dysfunction. Improved disease control may also reduce nocturnal symptoms, intermittent hypoxaemia, sleep disruption, and psychological distress. However, these potential neurocognitive effects remain hypothetical, and improvement in respiratory outcomes should not be assumed to translate directly into cognitive benefit.

### 10.5. Exploration of Targeted Interventions

The field should now move beyond observational associations towards intervention-based research. Optimising asthma control, minimising systemic corticosteroid exposure, treating sleep disorders and psychological comorbidities, and evaluating cognitive support or rehabilitation strategies represent promising approaches to preserving cognitive function.

Demonstrating that such interventions improve both respiratory and cognitive outcomes would provide important evidence that cognition represents not only a clinically meaningful consequence of asthma but also a potentially modifiable therapeutic target.

## 11. Conclusions

Asthma is increasingly recognised as a systemic disease with consequences that extend well beyond the airways, and accumulating evidence suggests that cognitive impairment may represent an under-recognised component of its clinical burden. Although the current evidence remains heterogeneous and does not establish causality, observational studies consistently demonstrate an association between asthma and impaired performance across multiple cognitive domains, particularly attention, executive function, memory, and processing speed. These deficits are likely to arise through the interaction of several disease-related mechanisms, including chronic systemic inflammation, intermittent hypoxaemia, sleep disturbance, corticosteroid exposure, and psychological comorbidity.

Even subtle cognitive dysfunction may have important clinical consequences by impairing self-management, medication adherence, symptom perception, and overall disease control, highlighting cognition as a clinically relevant outcome rather than simply an associated comorbidity. Establishing whether cognitive impairment represents a preventable or modifiable consequence of asthma should therefore become a priority for future research. Further investigation of cognitive health in asthma research and clinical practice may help inform risk stratification and personalised disease management, while broadening consideration of outcomes beyond traditional respiratory measurement.

## Figures and Tables

**Table 1:** Commonly used tools for the assessment of cognitive function.

Assessment Tool	Main Purpose	Key Cognitive Domains Assessed	Approximate Administration Time
Mini-Mental State Examination (MMSE)	General cognitive screening	Orientation, attention, memory, language, visuospatial skills	5–10 min
Montreal Cognitive Assessment (MoCA)	Screening for mild cognitive impairment	Attention, executive function, memory, language, visuospatial ability, orientation	10–15 min
Addenbrooke’s Cognitive Examination-Revised (ACE-R)	Comprehensive cognitive screening	Attention, memory, verbal fluency, language, visuospatial abilities	15–20 min
Clock Drawing Test (CDT)	Rapid cognitive screening	Executive function, visuospatial ability	2–5 min
Mini-Cog	Brief dementia screening	Memory recall, executive function	3–5 min
Informant Questionnaire on Cognitive Decline in the Elderly (IQCODE)	Informant-based assessment of cognitive decline	Functional and cognitive changes over time	5–10 min
Cambridge Cognitive Examination (CAMCOG)	Comprehensive cognitive assessment	Memory, attention, language, praxis, perception, executive function	20–30 min
Quick Cognitive Screening Test (QCST)	Rapid cognitive screening	Multiple cognitive domains	5–10 min
Memory and Executive Screening (MES)	Detection of mild cognitive impairment	Memory and executive function	5–10 min
Virtual Reality Cognitive Assessment (e.g., VR-based tools)	Technology-assisted cognitive assessment	Multiple cognitive domains in simulated environments	Variable

Abbreviations: MMSE, Mini-Mental State Examination; MoCA, Montreal Cognitive Assessment; ACE-R, Addenbrooke’s Cognitive Examination-Revised; CDT, Clock Drawing Test; IQCODE, Informant Questionnaire on Cognitive Decline in the Elderly; CAMCOG, Cambridge Cognitive Examination; QCST, Quick Cognitive Screening Test; MES, Memory and Executive Screening.

**Table 2:** Summary of Key Longitudinal Studies Examining Asthma and Cognitive.

Study	Population	Design	Cognitive Outcome	Main Finding
Ji et al., 2025 [41]	7948 adults; 329 developed asthmas	Longitudinal cohort; median follow-up 12.5 years	Memory decline	Memory decline accelerated after asthma onset (β = −0.029 SD/year; 95% CI: −0.049 to −0.010)
Christopher-Hayes et al., 2024 [42]	474 children	Longitudinal cohort; 2-year follow-up	Episodic memory	Earlier-onset asthma was associated with slower improvement in memory (β = −0.17; 95% CI: −0.28 to −0.05)
Bartels et al., 2024 [43]	5,460,732 Medicare beneficiaries	Retrospective cohort; 2-year assessment	Alzheimer’s disease	Asthma was associated with higher incident Alzheimer’s disease (adjusted OR = 1.33; 95% CI: 1.29–1.36)

## Data Availability

No new data were created or analysed in this study. Data sharing is not applicable.
